# Automated synthesis of radiopharmaceuticals for PET: an apparatus for [1-^11^C]labelled aldoses

**DOI:** 10.1155/S1463924694000246

**Published:** 1994

**Authors:** Shintaro Nishimura, Kazuyoshi Yajima, Norihiro Harada, Yasuhiro Ogawa, Nobuyoshi Hayashi

**Affiliations:** Institute for Biofunctional Research c/o National Cardiovascular Center 7-1-5-Chome Fujishiro-Dai, Suita City Osaka 565 Japan

## Abstract

This paper describes an instrumentation system for positron emission tomography (PET). A variety of [1-^11^C]labelled aldoses, such as [1-^11^C]-D-glucose, and galactose by a modification of the Kiliani-Fischer method have been produced. The instrumentation is fully automatic and consists of a synthesis system and control system. The synthesis system has the following functions: supplying reagents; performing reactions; purifying ^11^C labelled aldose; and preparing an injectable solution of ^11^C labelled aldose. These operations are performed by the control system in a remote control room. In a preliminary, hot experiment an injectable solution of [1-^11^C]-D-glucose was obtained. In addition, the operator is exposed to minimal radiation. The radioactivity of [1-^11^C]-Dglucose was 47 MBq, and the preparation time was 49 min.


Journal of Automatic Chemistry, Vol. 16, No. 6 (November-December 1994), pp. 195-204

### Automated synthesis of radiopharmaceuticals for PET: an apparatus for [1-$^{11}$C]labelled aldoses

**Shintaro Nishimura, Kazuyoshi Yajima, Norihiro Harada, Yasuhiro Ogawa and Nobuyoshi Hayashi**
*Institute for Biofunctional Research, c/o National Cardiovascular Center, 7-1-5-Chome, Fujishiro-Dai, Suita City, Osaka 565, Japan*

*This paper describes an instrumentation system for positron emission tomography (PET). A variety of [1-$^{11}$C]labelled aldoses, such as [1-$^{11}$C]-D-glucose, and galactose by a modification of the Kiliani-Fischer method have been produced. The instrumentation is fully automatic and consists of a synthesis system and a control system. The synthesis system has the following functions: supplying reagents; performing reactions; purifying $^{11}$C labelled aldose; and preparing an injectable solution of $^{11}$C labelled aldose. These operations are performed by the control system in a remote control room. In a preliminary, hot experiment an injectable solution of [1-$^{11}$C]-D-glucose was obtained. In addition, the operator is exposed to minimal radiation. The radioactivity of [1-$^{11}$C]-D-glucose was 47 MBq, and the preparation time was 49 min.*

### Introduction

Positron emission tomography (PET) [1] is a non-invasive imaging technique which can obtain biofunctional information from humans and animals using radiopharmaceuticals containing a positron emitter (for example $^{11}$C, $^{15}$O and $^{18}$F). There is currently great interest in the production of radiotracers for PET. [1-$^{11}$C]labelled aldoses are very useful radiotracers for regional cerebral glucose metabolism [2] and tumor markers [3]. However, there are some major synthetic problems in their preparation. $^{11}$C has a radioactive half-life of only 20·4 min and decays with the evolution of X-rays (energy = 511 keV). Moreover, the synthetic scale has to be very small because only pico-mol order of $^{11}$C can be obtained by $^{14}$N(p, $\alpha$)$^{11}$C reaction using a cyclotron. To overcome these difficulties, the preparation process has to be rapid, reproducible and on a micro scale. So the development of a rapid, stereoselective reaction and automation of the process are very important and the authors have been working on this.

The first synthesis of [1-$^{11}$C]-D-glucose using the classical Kiliani-Fischer method was reported by Shiue *et al.* [4], and recently Schoeps *et al.* [5] reported the preparation of [1-$^{11}$C]-D-glucose from [$^{11}$C]-nitromethane using a Nef reaction. In these approaches, the final product is obtained as a mixture of [1-$^{11}$C]-D-glucose and mannose, and the ratio of D-glucose to mannose was reported to be from 0·25 to 0·5. More recently, Carmen *et al.* [6] have improved the ratio of D-glucose to mannose by the reaction of D-arabinose with $NH_4{}^{11}CN$ in pH 8·1 borate buffer—the ratio was improved to 1·80 ± 0·57 in favour of D-glucose. Using these synthetic methods, several groups [7, 8] have developed remote or automated instruments for preparing [1-$^{11}$C]-D-glucose. However, the majority of these instruments were not fully automatic and were not very flexible. This paper describes a new method for preparing optical isomers by changing the reaction conditions and a new instrument set up which is fully automatic and can synthesize other [1-$^{11}$C]labelled aldoses.

### Method

### *Development of a rapid synthetic method*

Micro-scale synthetic study of aldoses was performed using a mock-up apparatus and cold experiments. The focus was on a modification of the Kiliani-Fischer method. Cyanohydrin formation with sodium cyanide and 2,3:4,5-di-*O*-isopropylidene-D-arabinose (1) [9] was investigated, and the optimum reaction conditions for preparing 3,4:5,6-di-*O*-isopropylidene-D-glucononitrile (2) [10] or mannononitrile (3) [10] by HPLC analysis were determined. Each aldononitrile was then converted to D-glucose or mannose by reductive hydrolysis with Raney nickel. In a similar manner, rapid synthetic methods for other aldoses were also investigated.

### *Construction of automated instrumentation*

The automated instrument was built for the synthetic method. To minimize the operator's exposure to radiation, the hardware was designed to produce an injectable solution through a remote control. The apparatus was designed for laboratory use and for ease of improvement of the hardware and software. The software was programmed with Hyakuninriki (Asahi Electronics Co. Ltd, Japan) which operates under MS-DOS.

### *Hot experiment*

After examining the synthesis of the aldoses in the cold test, an attempt was made to produce an injectable solution of [1-$^{11}$C]-D-glucose from $H^{11}CN$ [11] gas, which is prepared with a cyclotron and a $H^{11}CN$ gas generator—see figure 1. The production of $^{11}$C was accomplished by the nuclear reaction of accelerated protons with high pressured $^{14}N_2$ gas. This reaction was performed with a cyclotron and a target chamber. The pico mole quantities of $^{11}$C undergo rapid oxidation to $^{11}CO_2$ in the target chamber. The $^{11}CO_2$ gas was then transferred to the $H^{11}CN$ gas generator and converted to

0142-0453/94 $10.00 © 1994 Taylor & Francis Ltd.

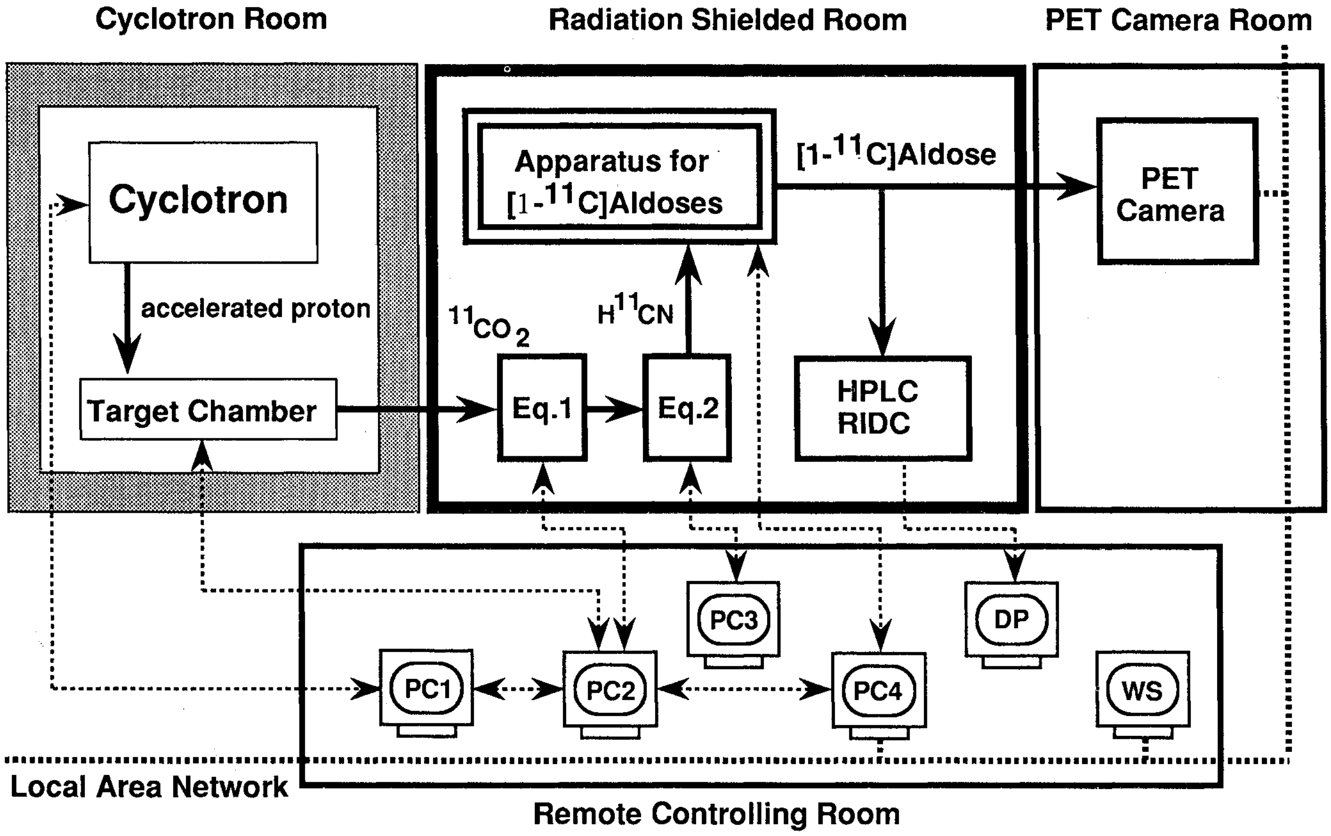

*Figure 1. Diagram of the production system for [1-$^{11}$C]labelled aldoses. PC1 = personal computer (PC-9801RA, NEC) for controlling a cyclotron (HM-18, SHI), PC2 = personal computer (PC-9801FA, NEC) for controlling Eq. 1, PC3 = personal computer (PC-9801BX, NEC) for controlling Eq. 2, PC4 = personal computer (PC-9821Ap, NEC) for controlling the apparatus, DP = data processor (C-R4AX, Shimadzu), Eq. 1 = $^{11}CO_2$ gas concentration equipment (AMCT 01, NKK Corp.), Eq. 2 = $H^{11}CN$ gas generator (AMMC 01, NKK Corp.), RIDC = radioisotope dose calibrator, WS = work station (SPARK station 10, SUN microsystems).*

$H^{11}CN$ gas as shown below:

$$^{14}N(p,\alpha)^{11}C \xrightarrow{O_2} {}^{11}CO_2 \xrightarrow[Ru / SiO_2]{H_2} {}^{11}CH_4 \xrightarrow{NH_3,\ Pt} H^{11}CN$$

1

Using the $H^{11}CN$ gas as the starting material, the automated synthesis of [1-$^{11}$C]-D-glucose was attempted. These operations were performed with four personal computers, and could be monitored with CCD video cameras in the remote controlling room. The product was analysed with a radioisotope dose calibrator and a HPLC system by remote monitoring and controlling.

### Results and discussion

### *Chemistry*

In order to investigate the possibility of selectively synthesizing either isomer by changing reaction conditions, reaction rate and the steroselectivity of cyanohydrin formation was examined. As a typical example, the reaction of compound 1 with one equivalent of sodium cyanide in a mixture of organic solvent and alkali buffer was chosen:

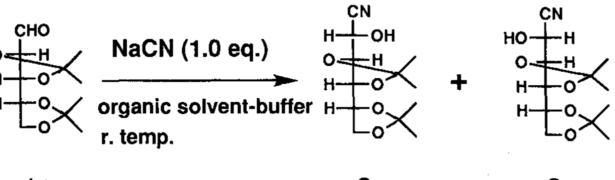

1 2 3

2

The yields of the cyanohydrins, gluconotrile 2 and mannononitrile 3, were measured using HPLC. The total yield curve producing 2 and 3 versus reaction time is shown in figure 2. The initial reaction rate calculated from the summation yield of 2 and 3, depended upon the organic solvent. However, the total yield curves appeared to level off within 5 min. Interestingly, the formation ratio of 2 to 3 was found to be greatly dependent on the organic solvent and the pH of the buffer—figure 3.

These results can be divided into three groups. First is the glucononitrile 2 selective group, in which a mixture of toluene and alkali buffer was used as the reaction solvent. In these cases, the formation of 2/3 increased with the reaction time and the pH value of the buffer, and levelled off after 5 min. Second is the non-selective group,

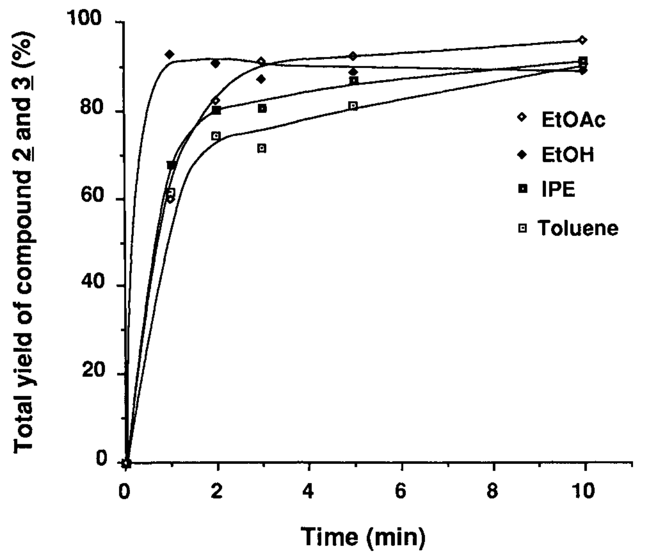

*Figure 2. Time course of total yield of compounds 2 and 3 in organic solvent and pH 10·8 buffer.*

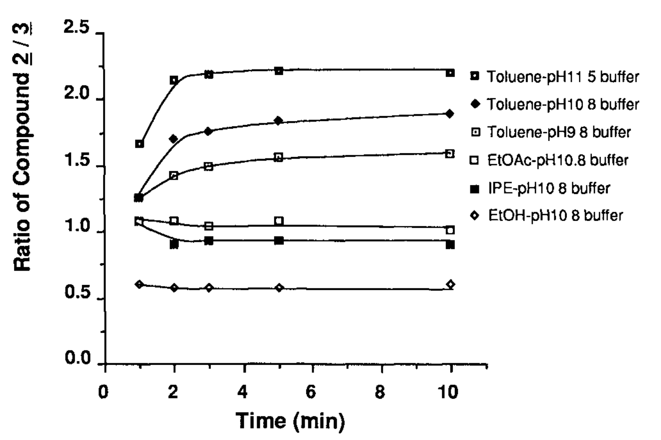

*Figure 3. Time course of the formation ratio of compound 2 to 3.*

in which a mixture of ethyl acetate or diisopropyl ether and alkali buffer was used. Last is the mannononitrile 3 selective group, in which a mixture of ethanol and alkali buffer was used. The formation ratio reached the steady state after 2 min. It seems that the increasing ratio with time is caused by equilibrium reaction between 2 and 3, and that the formation ratio of 2/3 relates to the polarity of the organic solvent.

In addition, the toluene-buffer condition for preparing 2, which is the precursor of D-glucose, was examined. Figure 3 shows that the stereoselectivity of the cyanohydrin formation depends on the pH. Figure 4 shows the yield curves of 2 when the pH varied from 8·3 to 11·5, but the reaction rate tended to decrease at high pH such as pH 11·5. Thus, the optimum pH was found to be 10·8 in figure 4. To obtain a better understanding of the reaction mechanism, the dissociation rates of 2 and 3 were measured. After cyanohydrins 2 and 3 were isolated by silica gel chromatography, each sample (10 µmol) was added to a mixture of toluene (50 µl) and aqueous pH 10·8 buffer (1 M $Na_2CO_3$–1 M HCl, 50 µl). Each mixture was stirred at room temperature and analysed by HPLC

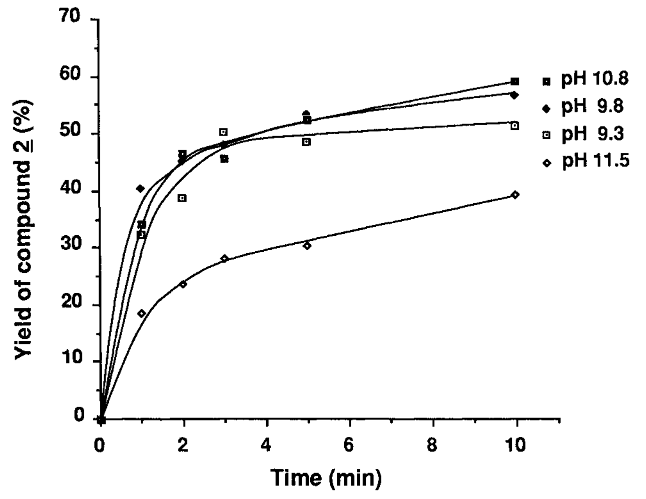

*Figure 4. Time course of yield of compound 2 in toluene-buffer (pH 9·3–11·5).*

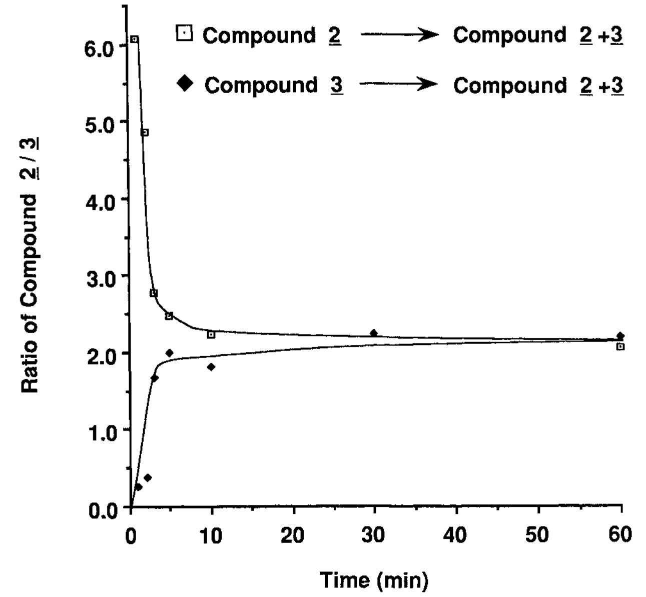

*Figure 5. Dissociation of compounds 2 and 3 in toluene-pH 10.8 buffer.*

as shown in figure 5. In both cases, the ratios of 2/3 varied with reaction time and after 5 min the ratios stabilized to 2·1 : 1. From these results, it is considered that the stereoselectivity of the cyanohydrin formation is not a result of cyanide attack on aldehyde 1, but, rather, it is due to an equilibrium reaction between the products 2 and 3. For the carbon-11 labelling, it was favourable that the equilibrium reaction proceeds rapidly. This method is practical and not moisture sensitive, so it is possible to apply to a microsynthesis of aldoses by combining it with a reductive hydrolysis step. Thus, a one-pot synthesis of D-glucose and mannose was as shown below:

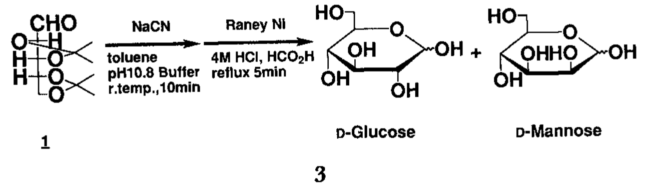

1

3

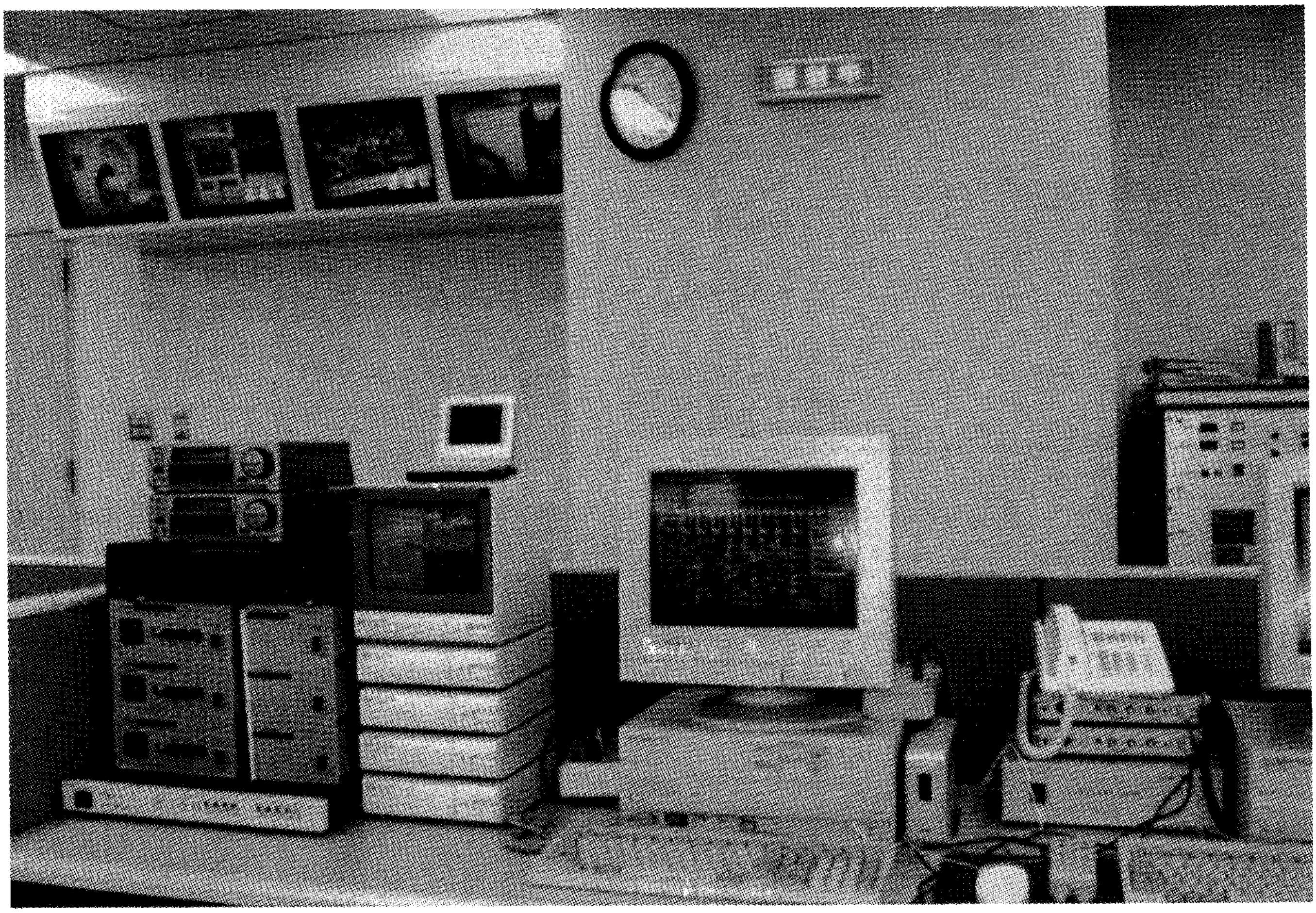

*Figure 6. General view of the remote control room.*

to give them in 23·0% and 13·5%, respectively. The one-pot reaction was performed within 15 min. Furthermore, we synthesized D-galactose (28·1%) and D-talose (11·9%) using a similar method from 2,3:4,5-*O*-isopropylidene-D-lyxose (4) [12]:

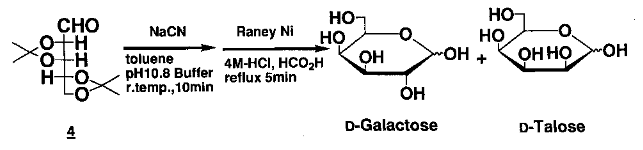

4

### Automated apparatus for [1-$^{11}$C]labelled aldoses

*General concept for the hardware*

The automated apparatus consists of the synthesis system and the controlling system. The synthesis system, an auto-manual switch box, and an interface are placed in a radiation shielded room. As these are removable, it is convenient for the cold experiment to be performed elsewhere and to facilitate the maintenance for the apparatus. The computer and its accessories are placed in the remote control room. The general appearance is shown in figures 6 and 7. The synthesis system consists of a series of units, which have the following functions: supplying reagents; performing reactions; purifying [1-$^{11}$C]labelled aldose; and preparing an injectable solution of [1-$^{11}$C]labelled aldose. These operations are performed by the controlling system and can be performed manually through the auto-manual switch box, which is useful in the case of the investigation with the cold experiment and the maintenance of the apparatus. As the solenoid valves and other devices of the reagents' supply unit and reaction unit were installed on the punched metal board, it is easy to modify the hardware.

*Synthesis system*

*Reagent and wash solvent supply unit*

The reagent supply unit [13] has eight reservoirs (12–19) in figure 8) for liquid reagents and solvents. Each reagent and solvent in the reservoirs is under nitrogen atmosphere, and can be transferred to the reaction flasks in two steps. First, the liquid is allowed to flow from the reservoir into a volumetric tube (0·5 ml) by nitrogen gas pressure. When the tube is full and the photosensor (45–52 in figure 8) is activated, the contents of the volumetric tube are emptied into the reaction flask by nitrogen gas pressure. The same volume of liquid may be repeatedly measured and added to the reaction flask. These operations are performed with three-way solenoid valves, photo-

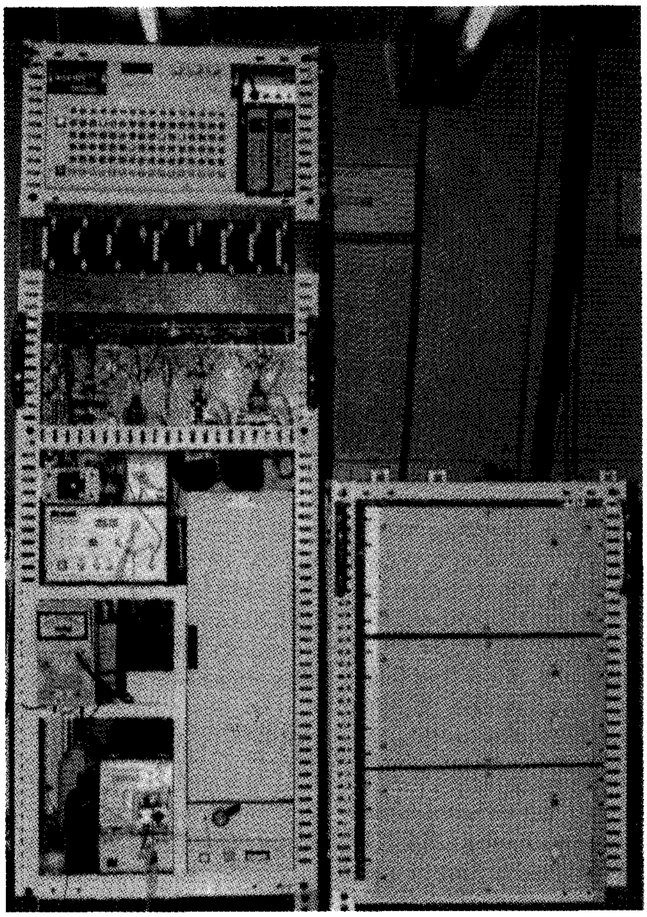

*Figure 7. Automated synthesis apparatus for [1-$^{11}$C]aldoses. The apparatus consists of two racks, the synthesis units and I/O boxes.*

sensors and nitrogen gas pressure. In this system, even moisture or air sensitive liquids can be stored in the reservoirs and transferred to the reaction flasks. After a synthetic run, all of the flow lines can be washed and dried by passing wash solvents which are stored in tanks (10 and 11 in figure 8), and then nitrogen gas through them.

### *Reaction unit*

The reaction unit has two reaction flasks (20 and 21 in figure 8). Reaction flask 1 is used for the hydrocyanation of a precursor with $Na^{11}CN$ and flask 2 is used for the reductive hydrolysis reaction. Both flasks are about 2 ml in volume and have jackets through which heating/cooling fluid is circulated with circulator 1 and 2 (24 and 25 in figure 8). The reaction temperature is maintained at the desired setting by the circulators. The mixing of the reaction mixture in the flasks are accomplished by nitrogen gas bubbling. The bubbling rate can be controlled with two mass flow controllers 1 and 2 (34 and 35 in figure 8). A reaction mixture in flask 1 can be transferred to flask 2 by using nitrogen gas pressure. The reaction mixture in flask 2 can be filtered with a glass filter, which is at the bottom of flask 2, and the filtrate can be transferred to the purification unit by using reduced pressure.

### *Purification unit*

The purification unit consists of three devices: an ion exchange resin column (29 in figure 8), an evaporating device, and a preparative HPLC device. The filtrate from

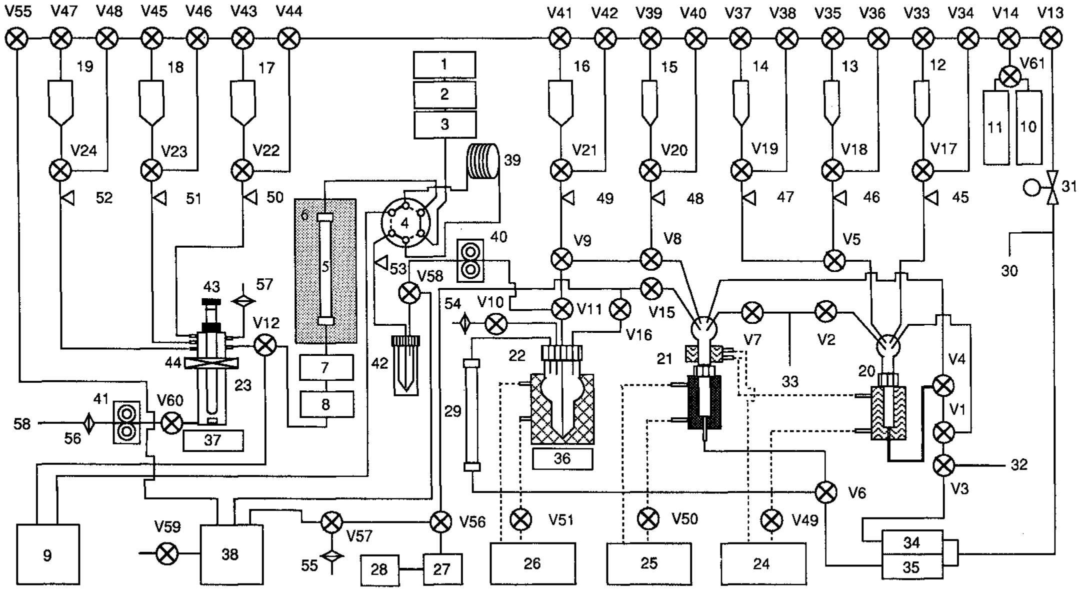

*Figure 8. Diagram of the synthesis system: V1–V61 = solenoid valves; 1 = tank of HPLC; 2 = degas device; 3 = HPLC pump; 4 = rotary 6-way valve; 5 = HPLC column; 6 = column oven; 7 = radiation detector; 8 = refractive index detector; 9 and 38 = drainage tanks 1 and 2; 10 and 11 = washing solvent tank 1 and 2; 12–19 = reservoirs 1–8; 20–23 = flasks 1–4; 24–26 = circulators 1–3; 27 = cold trap; 28 = vacuum pump; 29 = ion exchange resin column; 30 = nitrogen gas line; 31 = gas regulator; 32 = $H^{11}CN$ gas line; 33 = waste gas line; 34 and 35 = mass flow controller 1 and 2; 36 and 37 = magnetic stirrers 1 and 2; 39 = sample loop; 40 and 41 = roller pumps 1 and 2; 42 = bubble trap; 43 = pH sensor; 44 = level sensor; 45–53 = photosensors 1–9; 54–57 = filters 1–4; 58 = product output line; 59 = pressure sensor.*

flask 2 is desalted with the resin column and transferred to flask 3 for evaporation. Flask 3 also has a jacket through which heating fluid is circulated with circulator 3 (26 in figure 8), and is about 10 ml in volume. The mixing process in flask 3 is performed with the magnetic stirrer 1 (36 in figure 8). The evaporating process is carried out with a vacuum device (27 and 28 in figure 8). The concentrated mixture is then transferred to the auto-injection device through the bubble trap (42 in figure 8) with the roller pump 1 (40 in figure 8). An objective compound is isolated from the HPLC column (5 in figure 8) and detected with the refractive index and radiation detector (7 and 8 in figure 8). The eluate containing the objective compound is injected into the flask 4 (23 in figure 8).

*Pharmaceutical preparation unit*

The pharmaceutical preparation unit has three functions: adjusting the pH of the radiopharmaceutical solution; diluting with saline; and filtration with the filter 3 (56 in figure 8). The aqueous solution of the radiopharmaceutical in flask 4 is neutralized with a dilute acid or alkali solution from the reagent's supply unit. Flask 4 is equipped with the pH sensor (43 in figure 8), the level sensor (44 in figure 8), and the magnetic stirrer 2 (37 in figure 8). The radiopharmaceutical solution in flask 4 is filtered with the membrane filter and roller pump 2 (41 in figure 8). In the way, the $^{11}C$-labelled compound is available in ready-to-use form for the PET study.

*Control*

*Computer and software*

The instrumentation is controlled with a personal computer (PC-9821Ap, NEC), which is linked with the other computers and LAN (Local Area Network) as shown in figure 1. An OPTMUX (Opto 22, USA) interface unit is used. The computer software was developed by using Hyakuninriki. The program consists of four processes as follows; hydrocyanation process; reductive hydrolysis process; purification process; and pharmaceutical preparation process. A flowchart of these processes is shown in figure 9.

The hydrocyanation process contains subroutines from 'Add NaCN soln.' to 'Hydrocyanation in F1'. The reductive hydrolysis process contains subroutines of 'Add HCl HCOOH' and 'Reductive hydrolysis in F2'. The purification process contains subroutine from 'Desalt' to 'HPLC', and the pharmaceutical preparation process contains subroutines from 'pH adjustment in F4' to 'Volume adjustment in F4'. The reaction processes are controlled by a time sequential method, and the injection process in HPLC is performed by sequential control using the signal of the photosensor. The processes of pH and volume adjustments are controlled by a closed-loop method.

The objective compound is automatically isolated by the following method. An HPLC chart of the reaction mixture in the case of the preparation for D-glucose is shown in

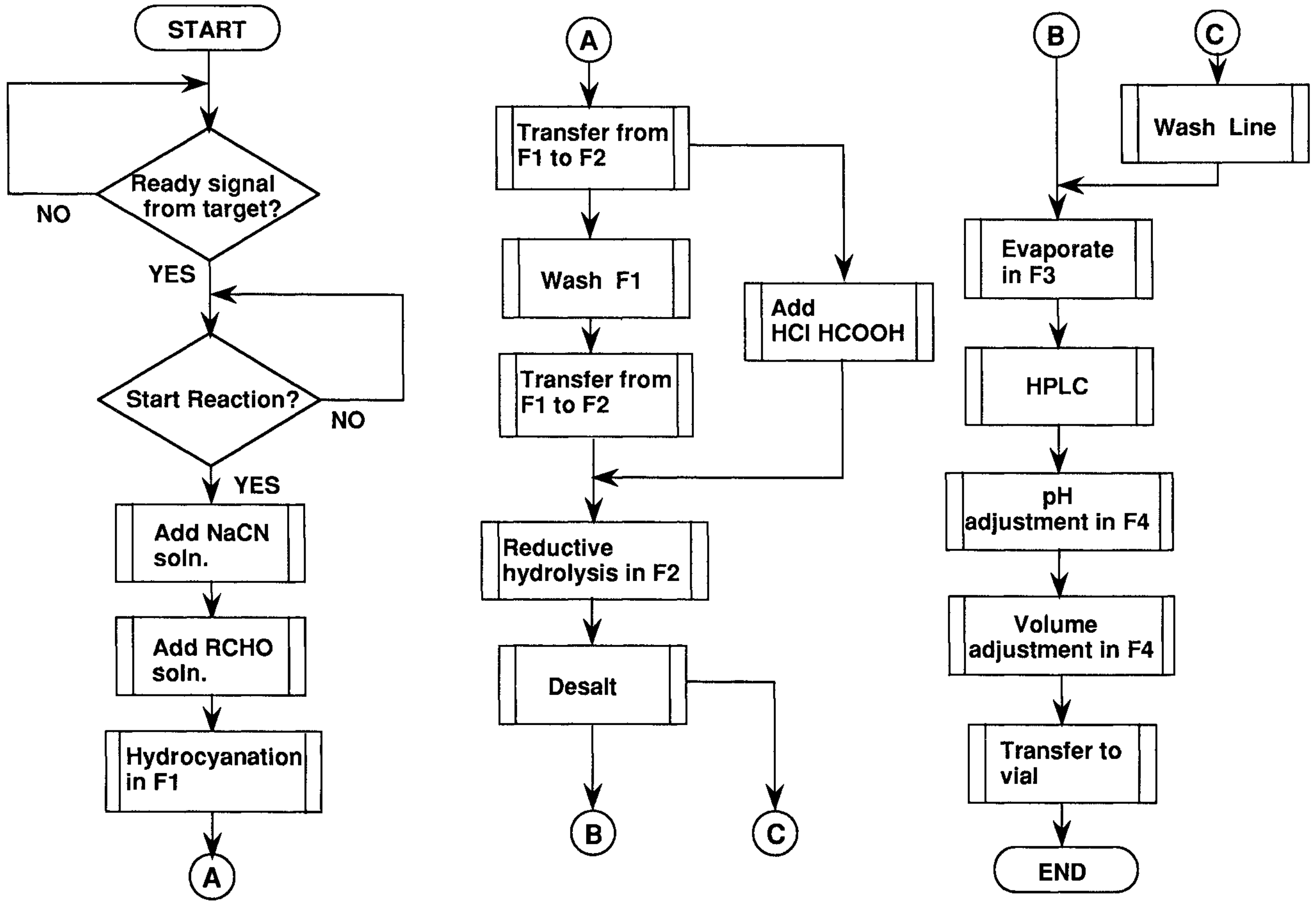

*Figure 9. Flowchart of the total operation. F1–4 = Flask 1–4.*

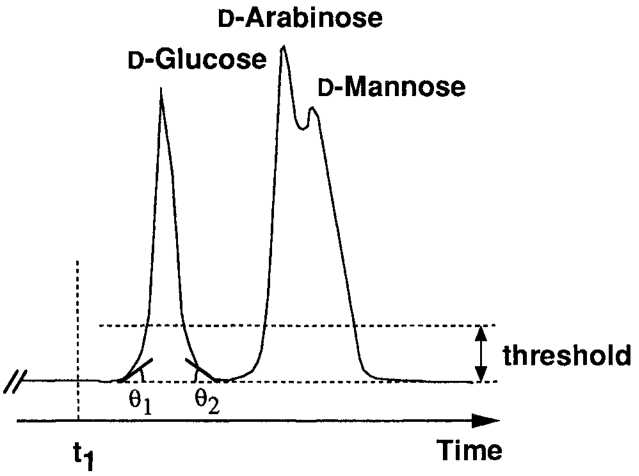

*Figure 10. HPLC chart of the reaction mixture detected with a refractive index detector.*

figure 10. Until the retention time comes to $t_1$ ($t_1$ = 12 min ~ 14 min), all the peaks of the HPLC are ignored. Collection of the eluate is started when the ratio of peak variation becomes $>\Theta_1$. Collection is stopped when the ratio of peak variation is $<\Theta_2$, and the peak value is less than the threshold value. This systematic procedure is highly efficient for isolating the objective compound even if the HPLC column becomes degraded. A flowchart of the operation for the 'HPLC' subroutine is given in figure 11.

### Automated synthesis of [1-$^{11}$C]-D-glucose

The instrumentation is able to produce a series of [1-$^{11}$C]labelled aldoses; among these, [1-$^{11}$C]-D-glucose is one of the most popular compounds for PET study. Therefore, an attempt was made to make an injectable solution of [1-$^{11}$C]-D-glucose.

### *Diagnosis check of the appararatus*

For radiation protection, a leak test on flasks 1 and 2 and their tube lines was performed by closing all outlets, opening them to the nitrogen gas flow line and monitoring the mass flow controllers. If zero flow could not be observed, the leak point was searched for and repaired until zero flow was eatablished.

### *Setting for a synthesis of [1-$^{11}$C]-D-glucose*

The HPLC system turned on, then the fluid of the circulators warmed up to the desired temperature (circulator 1: 25°C, circulator 2: 105°C, circulator 3: 80°C,

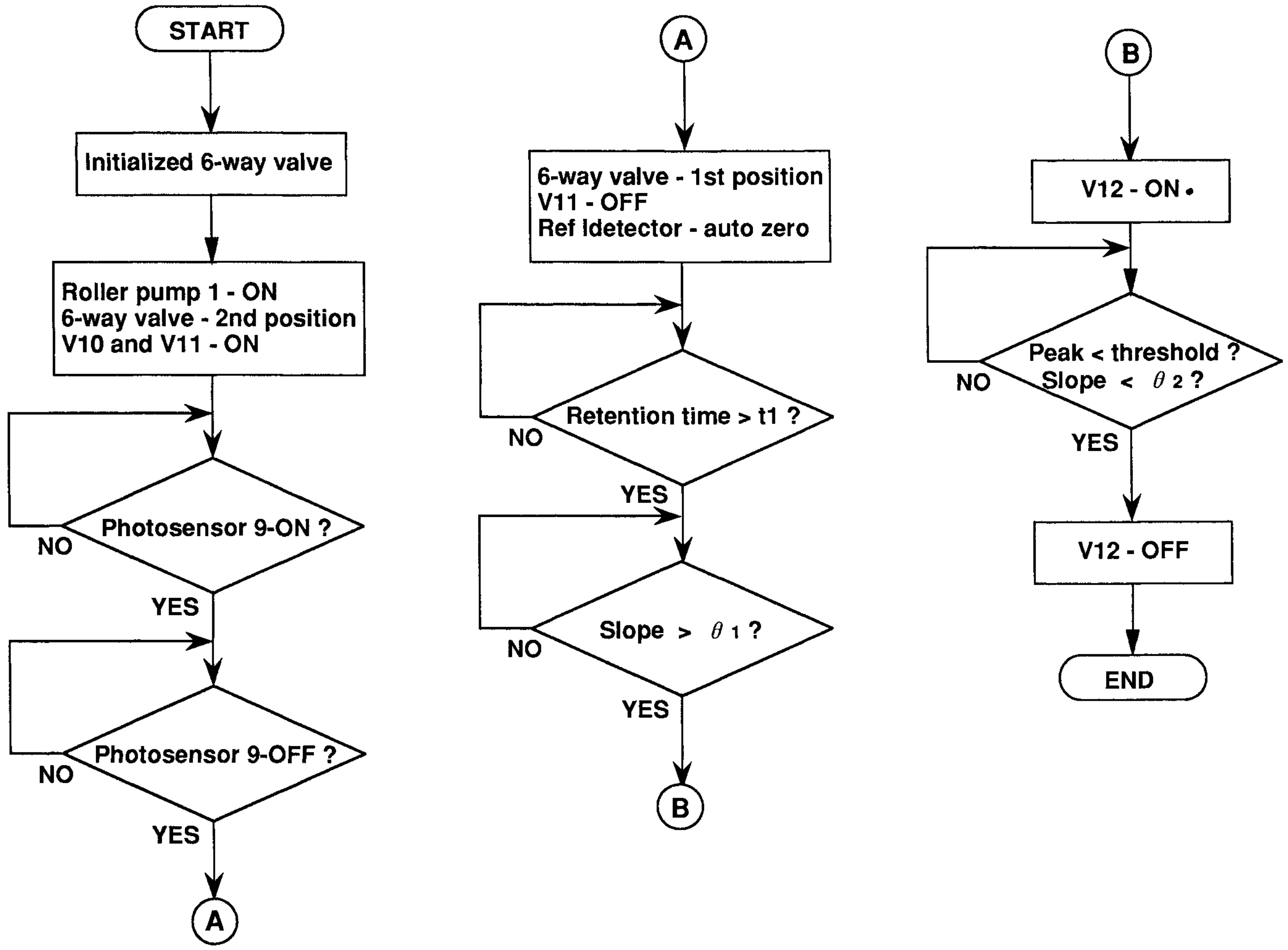

*Figure 11. Flowchart of the operation of the HPLC subroutine.*

24–26 in figure 8). The cold trap (27 in figure 8) for the vacuum pump had cooled to $-50°C$. The nitrogen gas regulator (31 in figure 8) was adjusted to 0·2–0·3 $kg/cm^2$. The reservoirs (12–19 in figure 8) of the apparatus were filled as follows: reservoir 1 (a mixture solution of NaCN (10 mg, as a carrier) and pH 10·8 buffer (1 M $Na_2CO_3$–1 M HCl, 1·0 ml)), reservoir 2 (a solution of compound 1 [46 mg] in toluene, 1·0 ml), reservoir 3 (toluene, 1·0 ml for washing solvent), reservoir 4 (a mixture of imidazole (40 mg), 4 M HCl (0·5 ml) and formic acid (0·5 ml)), reservoir 5 (distilled water, 10 ml for washing solvent), reservoir 6 (0·01 M HCl, 10 ml), reservoir 7 (0·01 M $NaHCO_3$, 10 ml), reservoir 8 (saline, 10 ml). The wash solvent tanks (10 and 11 in figure 8) were filled as follows: tank 1 (distilled water, 1000 ml), tank 2 (methanol, 1000 ml). Raney nickel (40 mg) was added to reaction flask 2 (21 in figure 8).

### *Synthesis of $H^{11}CN$*

The production of $H^{11}CN$ was accomplished by an on-line synthesis according to Iwata's method [11]. Production of $^{11}CO_2$ was accomplished through $^{14}N(p, \alpha)^{11}C$ reaction by proton bombardment (18 MeV, 15 μA) of 14·7 $kg/cm^2$ $N_2$ gas target using a cyclotron-target system (CYPRIS HM-18, Sumitomo Heavy Industries Co. Ltd). The $^{11}CO_2$ gas in the target chamber was transferred to $^{11}CO_2$ gas concentration equipment (AMCT 01, NKK Corp.) and then the concentrated $^{11}CO_2$ gas was transferred to an $H^{11}CN$ gas generator (AMHC 01, NKK Corp.) using He gas (flow rate: 100 ml/min) as a carrier gas, and hydrogenated (flow rate of $H_2$ gas: 10 ml/min) to give $^{11}CH_4$ gas at 200°C in the presence of silica-gel supported Ru catalyst. The reaction of $^{11}CH_4$ gas with $NH_3$ gas (flow rate: 5 ml/min) at 850°C in the presence of Pt catalyst gave off $H^{11}CN$ gas, which was passed through a $P_2O_5$ (5·0 g) column to remove excess $NH_3$ gas, and then 16·9 GBq (at the end of bombardment) of $H^{11}CN$ gas was transferred to the automated apparatus for [1-$^{11}$C]-labelled aldoses.

### *Hydrocyanation of compound 1*

Nitrogen gas flow rates of the mass flow controller 1 and 2 were set to zero. The outlet of circulator 1 was opened by switching the valve 49. The outlet of reservoir 1 was opened by switching the valves 17 and 33. A mixture solution of NaCN (as a carrier) and pH 10·8 buffer in reservoir 1 was allowed to flow from reservoir 1 into a volumetric tube (0·5 ml) by nitrogen gas pressure (nitrogen gas flow line: 30–31–V13–V14–V34–V33, in figure 8). When the tube was full and photosensor 1 activated, the contents of the volumetric tube were emptied into reaction flask 1 by switching the valve 17, 33, and 34 (nitrogen gas flow line: 30–21–V13–V14–V34–V17). When the volumetric tube was empty and the photosensor was off, the valve 34 was switched. Trapping of $H^{11}CN$ gas in flask 1 was carried out by switching valves 1, 3, and 4 ($H^{11}CN$ gas flow line: 32–V3–V1–V4-bottom of the flask 1). The waste gas was exhausted through valve 2 to the waste gas line. When the introduction of $H^{11}CN$ gas to flask 1 was finished, valve 3 was switched. To the mixture in flask 1 was added 0·5 ml of the solution of compound 1 in toluene from reservoir 2 in a similar manner to that of reservoir 1 using photosensor 2 and the valves 18, 35, and 36. The mixture in flask 1 was mixed for 8 min by nitrogen gas bubbling using the mass flow controller 1 (flow rate: 50 ml/min, flow line: 30–34–V3–V1–V4-bottom of the flask 1). When the hydrocyanation reaction was finished, the mixture in flask 1 was transferred to flask 2 by switching the valves 1 and 4 (nitrogen gas flow line: 30–34–V3–V1-top of the flask 1–V4-top of the flask 2). The flow rate of mass flow controller 1 was set to zero. The washing of flask 1 was performed with 0·5 ml of toluene from reservoir 3 and the washed solvent was transferred to flask 2 in a similar manner to that mentioned above.

### *Reductive hydrolysis of [1-$^{11}$C]aldononitrile*

A solution of formic acid, 4 M HCl, and imidazole (0·5 ml) from reservoir 4, was added to the mixture of the cyanohydrins and Raney nickel in flask 2. The outlet of circulator 2 was opened by switching valve 50. The mixture in flask 2 was mixed with nitrogen gas from mass flow controller 2 (nitrogen gas flow line: 30–35–V6-bottom of the flask 2) at a flow rate of 17·5 ml/min. The mixture was refluxed for 5 min. The waste gas was exhausted through valve 7 to the gas waste line. When the reaction was finished, the gas flow rate of the mass flow controller 2 was set to zero and valves 49 and 50 were closed to stop circulation.

### *Purification and pharmaceutical preparation of [1-$^{11}$C]-D-glucose*

The power switch of the vacuum pump and the magnetic stirrer 1 under flask 3 were turned on. The outlet of circulator 3 was opened by switching valve 51. When valves 6 and 16 were switched, the mixture was filtered with a glass filter fitted at the bottom of flask 2, the filtrate was passed through valve 6 and the ion exchange resin column (IRN-150L, 4·6 mm × 30 cm, Organo Co. Ltd) to flask 3. Evaporation of the contents of flask 3 was then performed by switching valve 6. While the evaporation was running, washing solvent (6·5 ml of water, 0·5 ml × 13 portions) from reservoir 5 was added to flask 2 (flow line: 16–V21–V9–V8-top of flask 2) in a similar manner, and then transferred to flask 3 through valve 6 and the column in the same manner. When the evaporation was finished, the outlet of circulator 3 was closed by switching valve 51, and in order to break the vacuum line valves 10, 16, amd 56 were switched and the power switch of the vacuum pump was turned off. The residue in flask 3 was dissolved in 0·5 ml water delivered from reservoir 5 in a similar manner (flow line: 16–V21–V9–V11-flask 3). After dissolving the residue, magnetic stirring was stopped, the aqueous solution was injected to the HPLC system as follows: valves 10 and 11 were switched, the aqueous solution containing air bubbles was delivered to the bubble trap *via* valve 58 with the roller pump 1, the debubbled solution in the bubble trap was sent to the sample loop *via* photosensor 9 and the rotary six-way valve, when the end of the solution line was detected by the photosensor 9, the rotary six-way valve was switched and the solution in the sample loop was loaded to the HPLC column.

The HPLC separation conditions were as follows: column: Bio-Rad Aminex HPX-87P (7·8 mm × 30 cm, 9 μm), mobile phase: water, flow rate: 0·6 ml/min, temperature: 85°C, retention time of D-glucose: 13·4 min. When the eluate containing [1-$^{11}$C]-D-glucose was detected with the refractive index detector (RI-71, Shodex Co. Ltd) and the radiation detector (TCS-R81–3454, Aloka Co. Ltd), valve 12 was switched and the eluate was transferred to flask 4. The magnetic stirrer was switched on and the pH value of the eluate in flask 4 was measured with the pH sensor. As the pH value was in the range from 6·5 to 7·5, the addition of the acid in reservoir 6 or the alkali in reservoir 7 was not performed. Thus the solution was diluted with the saline in reservoir 8. The saline was added to flask 4 repeatedly until the level sensor was activated which was set to a volume of 10 ml. Finally, the injectable solution of [1-$^{11}$C]-D-glucose was obtained by filtration with roller pump 2 and the membrane filter 3. The total synthesis time was 49 min. The product was analysed by remote monitoring and operating and the analysis data were found to be as follows: chemical yield 2·0% from NaCN, radiochemical yield 1·3% from $H^{11}CN$, radioactivity of [1-$^{11}$C]-D-glucose 47 MBq, chemical purity: >98%, radiochemical purity >95%.

### Conclusion

A rapid synthesis of aldononitrile compounds using an equilibrium reaction has been developed and the results show the feasibility of synthesis of either 2*R* or 2*S* aldoses under simple reaction conditions. On the basis of these investigations, an automated synthesis instrument was built which is capable of producing a wide variety of [1-$^{11}$C]labelled aldoses in a ready-to-inject form. As the instrumentation consists of a series of units and can be improved, it may be appropriate for laboratory use.

A preliminary, hot experiment using the instrumentation was successful and an injectable solution of [1-$^{11}$C]-D-glucose was obtained automatically. The difference in yield between the cold experiment and the hot experiment could be caused by the absorption in the flasks and columns. The authors are now working on the optimization of the operation conditions and the synthesis of the other labelled aldoses.

### Experimental

### *Materials and reagents*

Raney nickel was purchased from Nakarai Tesque, Inc. The other reagents and organic solvents were purchased from Wako Pure Chemical Industries, Ltd. All solvents were distilled and filtered with a membrane filter before use.

### *Analysis*

The melting-point of compound 2 is measured with a Yanagimoto micro melting-point apparatus without correction. NMR were recorded using Varian Instruments' GEM-300 spectrometer. Chemical shifts ($\delta$) were recorded in ppm from tetramethylsilane (in $CDCl_3$ and $C_6D_6$) as an internal standard. HPLC analysis was performed with a Shimadzu LC-9A pump, a Shodex refractive index detector RISE-61, an Aloka positron detector TCS-R81-3454 and three separate analytic systems were used:

(1) Analysis of aldononitrile 2 and 3:
Column: Waters radialpak C-18 (8 mm × 10 cm, 5 μm).
Mobile phase: acetonitrile: 0·003 M $KH_2PO_4$ = 2:3.
Flow rate: 1·5 ml/min.
Temperature: 25°C.
Retention time: compound 2 (12·38 min), compound 3 (11·47 min).

(2) Analysis of aldoses:
Column: Bio-Rad Aminex HPX-87P (7·8 mm × 30 cm, 9 μm).
Mobile phase: water.
Flow rate: 0·6 ml/min.
Temperature: 85°C.
Retention time: D-glucose (13·40 min), D-mannose (17·20 min), D-arabinose (16·30 min), D-galactose (14·53 min); D-talose (31·19 min), D-lyxose (17·38 min).

(3) Analysis of aldoses:
Column: Shodex Ionpak KS-801 (8 mm × 30 cm).
Mobile phase: water.
Flow rate: 1·0 ml/min.
Temperature: 80°C.
Rentention time: D-glucose (8·20 min), D-mannose (8·70 min).
D-Arabinose (9·30 min).

The analysis of [1-$^{11}$C]-D-glucose was performed by remote control. The HPLC analysis was accomplished using a Shimadzu LC-9A pump, a Shodex refractive index detector RISE-61, an Aloka positron detector TCS-R81–3454, a handmade auto sampler, and a Shimadzu C-R4AX two-channel data processor. The radioactivity of the product was measured with a CAPINTEC CRC-712 dose calibrator.

### *Cold synthesis of aldose derivatives*

#### *Preparation of 3,4:5,6-di-O-isopropylidene-D-gluconononitrile (2) and 3,4:5,6-di-O-isopropylidene-D-mannononitrile (3)*

To a solution of compound 1 [9] (450 mg, 1·95 mmol) in toluene (10 ml) was added a mixture of sodium cyanide (96 mg, 1·95 mmol) and pH 10·8 buffer (10 ml, 1 M $Na_2CO_3$–1 M HCl) at 25°C. The mixture was stirred at the same temperature for 10 min. The organic layer was separated, dried with anhydrous sodium sulphate, and evaporated *in vacuo* to give colourless oil. The residue was purified by silica-gel column chromatography (Wako, Wakogel C-200, 25 g, dichloromethane:diethyl ether = 40:1) to give compound 2 (218 mg, 43·5% as crystals) and compound 3 (118 mg, 23·5% as a colourless oil), respectively. Compound 2: melting point 82–84°C; $^1$H NMR ($C_6D_6$) $\delta$ = 1·133 (3H, s, $CH_3$), 1·286 (3H, s, $CH_3$), 1·396 (3H, s, $CH_3$), 1·419 (3H, s, $CH_3$), 3·672 (1H, m, $J$ = 6·0 and 9·1 Hz, H-5), 3·771 (1H, dd, $J$ = 3·0 and 9·1 Hz, H-3), 3·878 (1H, dd, $J$ = 6·0 and 9·1 Hz,

H-6a), 3·982 (1H, q, $J = 9{\cdot}1$ Hz, H-6b), 4·178 (1H, t, $J = 9{\cdot}1$ Hz, H-4), 4·461 (1H, d, $J = 11{\cdot}3$ Hz, 1-OH), 4·633 (1H, dd, $J = 3{\cdot}0$ and 11·3 Hz, H-2); $^{13}C$ NMR ($C_6D_6$) $\delta = 24{\cdot}772$ ($CH_3$), 26·316 ($CH_3$), 26·523 ($CH_3$), 26·920 ($CH_3$), 61·897 (C-2), 67·684 (C-6), 75·939 (C-5), 78·789 (C-4), 80·244 (C-3), 110·766 (isopropylidene, C), 118·445 (C-1). Compound 3: $^1H$ NMR ($CDCl_3$) $\delta = 1{\cdot}355$ (3H, s, $CH_3$), 1·431 (3H, s, $CH_3$), 1·441 (3H, s, $CH_3$), 1·471 (3H, s, $CH_3$), 3·763 (1H, dd, $J = 7{\cdot}9$ and 8·5 Hz, H-4), 4·010 (1H, dd, $J = 4{\cdot}3$ and 8·4 Hz, H-6a), 4·070 (1H, m, $J = 4{\cdot}3$, 5·7, and 8·5 Hz, H-5), 4·120 (1H, dd, $J = 5{\cdot}1$ and 7·9 Hz, H-3), 4·192 (1H, dd, $J = 5{\cdot}7$ and 8·4 Hz, H-6b), 4·588 (1H, d, $J = 5{\cdot}1$ Hz, H-2); $^{13}C$ NMR ($C_6D_6$) $\delta = 25{\cdot}043$ ($CH_3$), 26·208 ($CH_3$), 26·679 ($CH_3$), 26·942 ($CH_3$), 63·516 (C-2), 67·799 (C-6), 76·271 (C-5), 79·292 (C-4), 80·577 (C-3), 109·98 (isopropylidene, C), 111·83 (isopropylidene, C), 117·496 (C-1).

*One-pot synthesis of* D*-glucose and* D*-mannose*

A mixture of sodium cyanide (5 mg, 0·1 mmol) and pH 10·8 buffer (0·5 ml, 1 M $Na_2CO_3$–1 M HCl) at 25°C was added to a solution of compound 1 (23 mg, 0·1 mmol) in toluene (0·5 ml). The mixture was stirred at the same temperature for 10 min. To the mixture was added a mixture of Raney nickel (40 mg), formic acid (0·25 ml), 4 M HCl (0·25 ml), and imidazole (20 mg). The mixture was heated at 105°C for 5 min, and then filtered. The filtrate was evaporated *in vacuo* to give a residue. The purification was performed by the HPLC system to give D-glucose (4·1 mg, 23·0%) and D-mannose (2·4 mg, 13·5%), respectively. These operations were carried out using the mock-up apparatus.

*One-pot synthesis of* D*-galactose and* D*-talose*

These compounds were prepared in a similar manner to that of D-glucose and D-mannose. The starting material, 2,3:4,5-di-*O*-isopropylidene-D-lyxose (4) can be derived by Lee's method [12]. In this way, D-galactose and D-talose were obtained in yields of 28·1% and 11·9%, respectively.

### Acknowledgements

The authors wish to thank Dr Teruo Omae, President of the National Cardiovascular Center, for supporting this work, and to Drs Masadzumi Watanabe, Tohru Sugawara, and D. G. Cork at Takeda Chemical Industries Ltd for useful discussions.

### References

1. Wolf, A. P. and Flower, J. S., in *Positron Emission Tomography*, Reivich, M. and Alavi, A. (Eds) (Alan R. Liss Inc., New York, 1985), 63.
2. Raichle, M. E., Larson, K. B., Phelps, M. E., Grubb, Jr., R. L. and Ter-Pogossian, M. M., *American Journal of Physiology*, **228** (1975), 1936.
3. Bergstrom, M., Collins, P. and Ehrin, E., *Computer Assisted Tomography*, **7** (1983), 1062.
4. Shiue, C.-Y. and Wolf, A. P., *Journal of Labelled Compounds and Radiopharmaceuticals*, **22** (1985), 171.
5. Schoeps, K.-O., Långström, B., Stone-Elander, S. and Halldin, C., *Applied Radiation and Isotopes*, **42** (1991), 877.
6. Dence, C. S. and Welch, M. J., *Journal of Labelled Compounds and Radiopharmaceuticals*, **32** (1993), 574.
7. Links, J. M., Courter, J. H. and Krohn, K. A., *Journal of Nuclear Medicine*, **30** (1989), 928.
8. Stone-Elander, S., Halldin, C., Långström, B., Blomqvist, G. and Widen, L., *Applied Radiation and Isotopes*, **43** (1992), 721.
9. Zimmer, H., Wittenburg, E. and Rembarz, G., *Chemische Berichte*, **92** (1959), 721.
10. Nishimura, S. and Hayashi, N., *Chemistry Letters (Japan)* (1991), 1815.
11. Iwata, R., Ido, T., Takahashi, H. and Iida, S., *Applied Radiation and Isotopes*, **38** (1987), 97.
12. Lee, A. W. M., Martin, V. S., Masamune, S., Sharpless, K. B. and Walker, F. J., *Journal of American Chemical Society*, **104** (1982), 3515.
13. Hayashi, N., Sugawara, T., Shintani, M. and Kato, S., *Journal of Automatic Chemistry*, **11** (1989), 212.

